# Fecal microbiome signatures of pancreatic cancer patients

**DOI:** 10.1038/s41598-019-53041-4

**Published:** 2019-11-14

**Authors:** Elizabeth Half, Nirit Keren, Leah Reshef, Tatiana Dorfman, Ishai Lachter, Yoram Kluger, Naama Reshef, Hilla Knobler, Yaakov Maor, Assaf Stein, Fred M. Konikoff, Uri Gophna

**Affiliations:** 1 Department of Gastroenterology, Rambam Health Care Campus, Haifa, Israel; 2 School of Molecular Cell Biology and Biotechnology, George S. Wise Faculty of Life Sciences, Tel Aviv University, Tel Aviv, Israel; 3 Department of General Surgery, Rambam Health Care Campus, Haifa, Israel; 4 Diabetes, Metabolic and Endocrinology Institute, Kaplan Medical Center, Rehovot, Israel; 5 Institute of Gastroenterology and Hepatology. Kaplan Medical Center, Rehovot, Israel; 6 Department of Gastroenterology and Hepatology, Meir Medical Center, Kfar Saba, Israel; 7 Sackler Faculty of Medicine, Tel Aviv University, Tel Aviv, Israel; 8 The Rappaport Faculty of Medicine, Technion, Haifa Israel

**Keywords:** Microbiome, Diagnostic markers

## Abstract

Pancreatic cancer (PC) is a leading cause of cancer-related death in developed countries, and since most patients have incurable disease at the time of diagnosis, developing a screening method for early detection is of high priority. Due to its metabolic importance, alterations in pancreatic functions may affect the composition of the gut microbiota, potentially yielding biomarkers for PC. However, the usefulness of these biomarkers may be limited if they are specific for advanced stages of disease, which may involve comorbidities such as biliary obstruction or diabetes. In this study we analyzed the fecal microbiota of 30 patients with pancreatic adenocarcinoma, 6 patients with pre-cancerous lesions, 13 healthy subjects and 16 with non-alcoholic fatty liver disease, using amplicon sequencing of the bacterial 16S rRNA gene. Fourteen bacterial features discriminated between PC and controls, and several were shared with findings from a recent Chinese cohort. A Random Forest model based on the microbiota classified PC and control samples with an AUC of 82.5%. However, inter-subject variability was high, and only a small part of the PC-associated microbial signals were also observed in patients with pre-cancerous pancreatic lesions, implying that microbiome-based early detection of such lesions will be challenging.

## Introduction

Pancreatic cancer (PC) is the 4th and 5th leading cause of cancer-related death in the USA and the EU, respectively^1,2^. In contrast to the reduction in the incidence of colon cancer due to screening and early detection, PC age-standardized rates have remained stable, suggesting that an improvement in life expectancy can be gained only through development of novel therapeutic approaches or through prevention\earlier detection^3^. As it is estimated that pancreatic cancer develops over longer than a decade^4,5^, a window of opportunity for early intervention does exist.

The main route of prevention\early detection of PC at present relies on advanced pancreatic imaging to detect pre-cancerous lesions^6,7^. As this method is difficult to implement in wide-range screening of large populations, considerable effort is being invested in identification of molecular, proteomic or metabolomic signatures in body fluids^8^, which may be used as biomarkers for early PC.

An additional strategy for development of precancerous markers is to identify alterations in the microbial populations resident in the human body, commonly referred to as “microbiomes”. Farrel *et al*.^9^ were first to demonstrate alterations in the oral microbiome of PC patients when compared to healthy controls; however, with a best-case specificity of 82.1%, this microbial signature, while impressive, was not strong enough for diagnosis of a rare-incidence disease such as PC. While the oral microbiome is probably only indirectly related to pancreatic function, the gut microbiome is directly affected by pancreatic secretions that fundamentally affect digestion and metabolism. Therefore, even minor changes to pancreatic tissue could cause alterations in intestinal metabolite concentrations, which are highly likely to be sensed by the intestinal microbial communities. As the gut microbiome can be easily profiled by a fecal sample on a semi-automated platform, this approach is appealing in its simplicity, and has been recently put forward by Ren *et al*.^10^, in a study comparing the fecal microbiome of PC patients and matched controls of Chinese origin. However, though a PC-associated fecal microbial signature was clearly demonstrated, there was no real improvement in its specificity and sensitivity compared to the oral signature shown by Farrel.

Taken together, these data suggest that while PC-associated microbial signatures are easily observed, their translation to predictive biomarkers is not straightforward. The human gut microbiome is highly variable between different subjects and strongly affected by environment^11^. Thus, while statistically significant microbial patterns characterizing particular patient groups are evident, their practical value for diagnostic purposes would depend upon several additional factors. First, the pattern should be robust in differentiating the condition of interest from a wide range of other conditions, not merely from healthy control subjects. In this regard, it should be kept in mind that PC patients often display co-morbidities, ranging from obesity, diabetes and pancreatitis, considered risk factors of the disease, to biliary obstruction which is a common consequence of the disease. Secondly, the pattern must be reasonably consistent across all patients with a specific clinical condition. Here, we must closely examine variations between individuals *within* the same cohort, as well as *between* study cohorts – since origin and geographic location, as well as personal dietary and lifestyle habits, impact the microbiome. Finally, the pattern should be evident in early disease stages; in the case of PC, an ideal biomarker should be evident in patients with pre-cancerous lesions, before the progression to incurable cancer.

In this study, we examined the gut microbiome alterations in PC and their potential to serve as biomarkers in an Israeli cohort. We compared the microbiome of PC patients to that of a pre-cancerous lesion group (PCL), a group composed of individuals with non-alcoholic fatty liver disease (NAFLD) and a healthy control group. We also associated the microbiome composition with a range of background clinical conditions, as well as serum biochemical markers for biliary obstruction and liver damage. Finally, we assessed microbial variance both within our cohort and between the Israeli and Chinese cohorts.

## Materials and Methods

### Subjects

Patients presenting with pancreatic cancer (n = 30) or pre-cancerous lesions (n = 6) were recruited among individuals who were seen at the surgical ward or GI clinics of Rambam Health Care Campus or Meir Medical Center. The diagnosis was verified by histological samples obtained by EUS or by postoperative pathological assessment. Pre-cancerous lesions (PCL) were defined by standard clinical criteria as a cystic lesion with dilated main pancreatic duct or side branch ducts; the PCL group consisted of 4 patients with low-grade Intraductal papillary mucinous neoplasm (IPMN), one with multifocal IPMN, and one with pancreatic intraepithelial neoplasia (panIN). Control subjects (n = 13) were recruited from healthy volunteers at Rambam Health Care Campus and from individuals undergoing screening colonoscopy at Meir Medical Center; individuals under the age of 50 were excluded from analysis. An additional control group was recruited from subjects with non-alcoholic fatty liver disease (NAFLD, diagnosed by ultrasonography and alanine aminotransferase levels of (ALT) ≥ 30 U/L for male and ≥19 U/L for females), seen at Kaplan Medical Center (n = 16). Individuals who were exposed to antibiotics up to 8 weeks before sampling, who had a history of prior cancer, pancreatitis, acute or chronic intestinal inflammation, or who carry known cancer-associated mutations were excluded from analysis. This study received the approval of the Rambam Health Care Campus and Meir Medical Center ethics committees (study approval number: 0345-12-RMB), and all tests were performed in accordance with the relevant guidelines and regulations. Each participant in this study provided written informed consent.

### Sample processing and DNA sequencing

Samples from PC, PCL and NAFLD patients were collected as soon as possible after diagnosis and prior to any treatment, and stored at the medical facility at −80 °C. Samples from healthy subjects undergoing colonoscopy were collected within two weeks and transported on ice to the medical facility, where they were stored as above. Samples were transported to the research facility on dry ice. Fecal samples were stored upon procurement at the medical institute at −80 °C. DNA was extracted using the PowerSoil™ DNA extraction kit (MOBIO) according to the HMP (Human Microbiome Project) guidelines. PCR amplification of the 16S rRNA gene was carried out with universal prokaryotic primers containing 5-end common sequences as previously described^12^ (CS1-341F 5′-ACACTGACGACATGGTTCTACANNNNCCTACGGGAGGCAGCAG and CS2-806R 5′-TACGGTAGCAGAGACTTGGTCTGGACTACHVGGGTWTCTAAT). Twenty-four PCR cycles (95 °C 15 sec., 53 °C sec. 15, 72 °C 15 sec) were conducted using the PCR mastermix KAPA2G Fast™ (KAPABiosystems); successful amplification was verified by agarose gel electrophoresis. Paired-end deep sequencing of the PCR products was performed on an Illumina MiSeq platform at the University of Illinois at Chicago Sequencing Core (UICSQC). Sequencing depth ranged from 1070 to 31118 sequences per sample; to ensure data evenness, data was initially rarefied to 3 optional sequences depths: 1070 (retaining all study samples), 3000 (discarding 3 samples), and 9000 (discarding 4 samples). Similar microbial patterns were observed across all three optional depths. Data was thus rarefied to 1070 seqs/sample, so as to retain all samples.

### Data analysis

Demultiplexed raw sequences were quality filtered (removing bases with a PHRED quality score < 20), length filtered (discarding sequences shorter than 380 bp) and merged using PEAR^13^. Data was then processed with a custom workflow combining the Quantitative Insights Into Microbial Ecology (QIIME) package^14^ and VSEARCH^15^, and according to the strategy described in the UPARSE pipeline^16^. In brief, amplification primers were removed and the data were converted to a single FASTA file using QIIME scripts. VSEARCH was used for dereplication and OTU picking at 99% identity; to reduce spurious OTU formation, only sequences that appeared more than 5 times (100% similarity) were allowed to form new OTUs. Chimeric OTUs,identified by UCHIME^17^ using the GOLD.fa database as reference, were removed. Centroid sequences of the remaining (non-chimeric) OTUs were then used as a database, against which all the sequences, including singletons, were mapped to form an OTU count table. Taxonomy assignment (using UCLUST algorithm^18^ against Silva v128 database), rarefaction, and UniFrac calculations were done using QIIME. Several well-established similarity indexes in microbial ecology (Bray-Curtis, Jaccard, abundance-weighted and unweighted UniFrac indexes) were used to calculate the distance between each pair of samples; the degree of separation between the microbiome of PC and all control samples was then assessed by the ANOISM probability test. Integration with data from the Ren *et al*.^10^ study was done utilizing taxonomical information at the OTU level. Briefly, we first reassigned taxonomy to the OTUs defined in our study using the RDP classifer, the method used in the Ren *et al*. study^10^. Within each study, we then collapsed OTUs which were assigned identical taxonomies to a single entity, preserving abundance information, and achieving, per study, an OTU abundance table containing unique taxonomy classifications. Both tables were then transformed to relative abundances (RA, by Total Sum Normalization, TSS), and finally merged by taxa names to a single RA table used for analysis as described below.

### Statistical analyses

R vegan^19^ package was used to calculate Shannon diversity index, Bray-Curtis/Jaccard distance matrices, and ANOSIM probability tests. PCoAs were constructed using the base R function cmdscale and plotted with ggplot2^20^ package. Hierarchical clustering of samples was conducted on each distance matrix using the hclust R function and UPGMA method, and the R package dendextend^21^ was used for the plotting. The randomForest^22^ and pROC^23^ packages were used for building classification models and plotting ROC curves. Kruskal-wallis statistical test, Spearman’s correlations and Benjamini-Hochberg^24^ corrections for multiple hypothesis testing were all conducted using base R functions; package dunn.test^25^ was used to compare values across multiple groups using Dunn’s test. P-values < 0.05 or q values < 0.2 were considered significant. LEfSe^26^ was applied to identify which bacterial taxa contribute to the differences between the two groups; p-value for the 1st (Kruskal-Wallis) step was set at 0.05, and LDA minimal threshold set at 3.

## Results

This study was conducted on a cohort composed of 30 patients with PC and two control groups: one of healthy subjects (n = 13), and a second of patients with non-alcoholic fatty liver disease (NAFLD, n = 16). As gut microbiome may be highly sensitive to liver function, which in turn is often impaired in advanced PC, individuals with NAFLD but no pancreatic disorders were used as an additional, stringent control group. Six additional patients were diagnosed with pre-cancerous pancreatic lesions (PCL). Patient information is summarized in Table 1; additional information on the stage of cancer, when available, is presented in Supplementary Table S1. Deep amplicon sequencing of the bacterial 16S rRNA gene was used to assess specific features in the microbial populations associated with each group; barplots showing detailed taxonomical composition across all four groups are provided in Supplementary Fig. S1. The differences in alpha diversity between PC, PCL and healthy control groups did not  reach statistical significance (median Shannon values: PC = 2.89, PCL = 3.04, Control = 3.1). However, the NAFLD group were slightly more diverse than all other groups (p = 0.01, median Shannon = 3.3, Supplementary Fig. S2).

**Table 1:** Patient information.

	PC	Non-alcoholic fatty-liver disease (NAFLD)	Pre-cancerous lesions (PCL)	Healthy
n	30	16	6	13
Age (mean+/−s.d.)	68.9 ± 6.2	51 ± 10.8	66 ± 15.3	59 ± 8.7
Sex (M/F)	16/14	12/4	5/1	6/7
Diabetes (%)	53	13	20	NA
High Blood Pressure (%)	43	50	25	NA
Bile-duct obstruction* (%)	36	0	0	0
Gall-bladder abnormalities* (%)	46	6	0	23
Hyperlipidemia (%)	40	88	29	23
**Biochemical Assays (serum**, **mean** ± **s**.**d**.^+^**)**				
Total Bl (mg/dl)	2.3 ± 2.8	0.6 ± 0.2	n.d.	
Direct BL (mg/dl)	1.7 ± 2.5	0.2 ± 0.1		
GGT (U/L)	474 ± 806	48 ± 28		
AST (U/L)	86 ± 103	31 ± 14.5		
ALT (U/L)	160 ± 199	50 ± 23		

^*^Dilated CBD (common bile duct) diameter on US or CT and\or elevated liver functions tests as GGT, AP and Bilirubin.

**Gall-bladder abnormalities included gallstones, thickened bladder wall, swollenness or prior removal.

^+^Differences in biochemical assay levels between PC and NAFLD were not statistically significant (p > 0.2 for all assays).

### Large scale differences in microbiome of PC patients and controls

**Figure 1:**
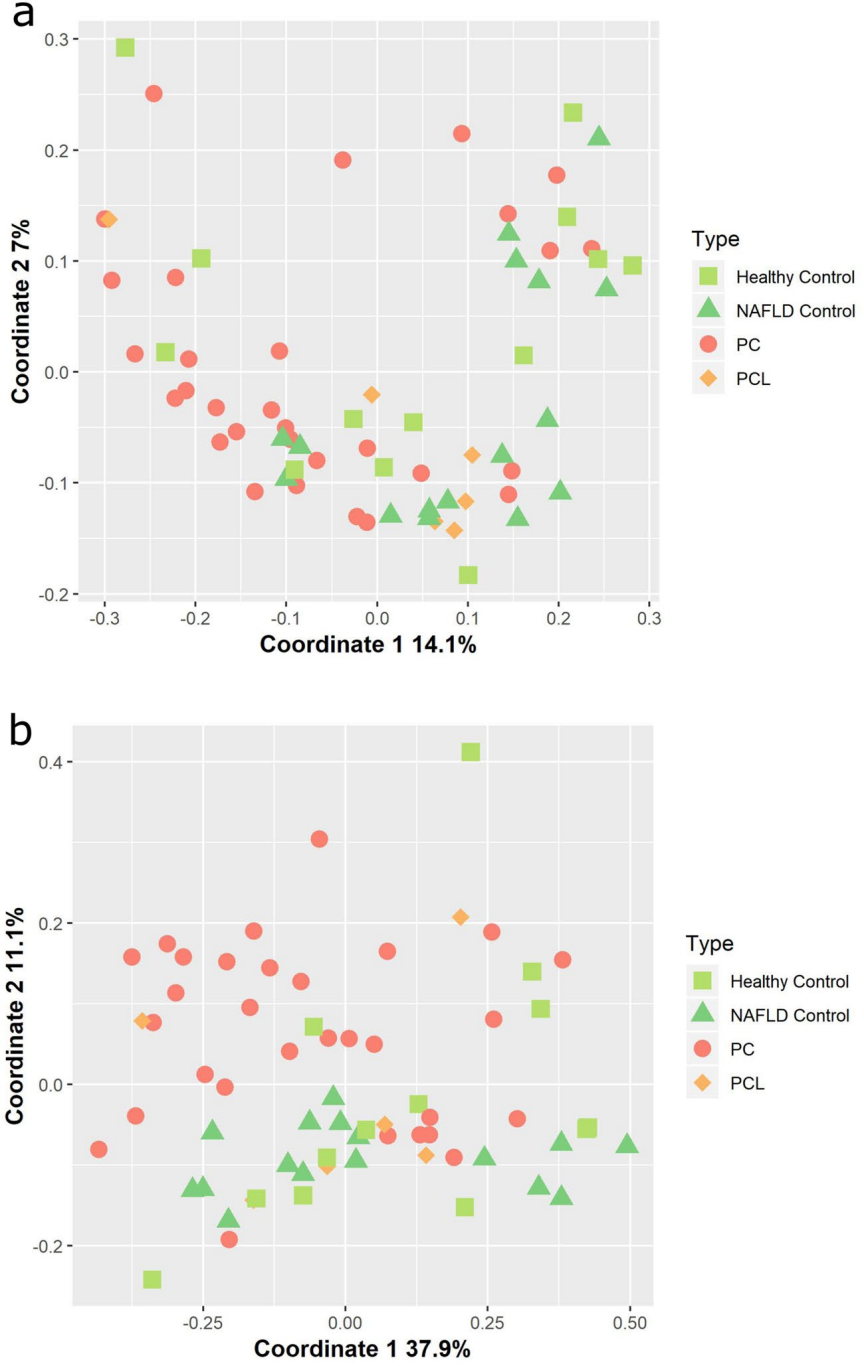
Similarity in microbial composition across different sample types. Principal Coordinate Analysis (PCoA) was used to visualize spatial relationships among samples according to (**a**) abundance-weighted UniFrac and (**b**) unweighted UniFrac distance matrices.

PC patients constitute a heterogeneous group, often suffering from a range of background conditions in addition to complications brought on by PC, such as diabetes (Table 1). Accordingly, the microbial composition of samples from PC patients was highly variable. Nevertheless, some universal changes in the microbiome of PC patients, when compared to that of both control (NAFLD or healthy) groups, were evident. The ratio between Bacteroidetes and Firmicutes, the two dominant bacterial phyla in the human gut, was higher in PC patients than in any of the control groups Supplementary Fig. S2). Additionally, a weak but significant degree of separation in microbial composition according to clinical status was observed when comparing PC to the healthy control group using the ANOSIM probability test (abundance-weighted UniFrac: p = 0.013, R = 15%; unweighted-UniFrac: p = 0.04, R = 13%). Principle Coordinate analysis (PCoA) was applied to generate a visual representation of this trend across all patient groups (Fig. 1). Noticeably, while some separation of PC samples from both healthy and NAFLD control groups is evident, there is also substantial overlap between these groups. This overlap, in agreement with the relatively low ANOSIM R values, implies the involvement of additional factors in determining gut microbial composition; this issue is discussed in detail below. The PCL group, too small to be included in statistical significance testing, appears to overlap both the PC and control groups (Fig. 1; see also Hierarchical clustering of samples in Supplementary Fig. S3).

### Specific microbial patterns associated with PC

**Figure 2:**
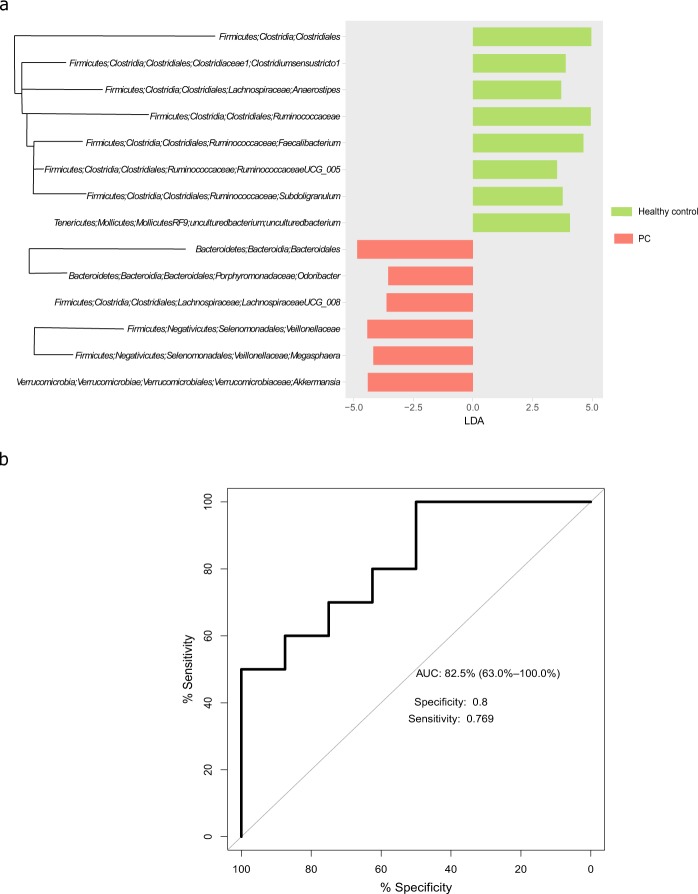
Microbial features characterizing PC. (**a**) Bacterial taxa identified by LEfSe as differentiating between PC patients (red) and healthy control subjects (green). Phylogenetically related bacterial taxa are denoted by connecting branches. (**b**) ROC curve of a random forest model, trained on the features identified in (**a**).

The well-established feature analysis tool, LEfSe [linear discriminant analysis (LDA) effect size]^26^, was used to identify features which were differentially distributed between PC patients and the healthy control group (Fig. 2). Features discriminating PC from healthy controls were evident at most phylogenetic levels. Considerable under-representation of bacterial orders, families and genera belonging to the Firmicutes phylum was observed in the PC group, thereby corroborating the trend of a decreased Firmicutes/Bacteroidetes ratio in PC patients, observed at the phylum level.

A comparison of PC to the NAFLD group yielded similar results, with multiple reductions in taxa belonging to the Firmicutes observed in PC relative to NAFLD (Supplementary Fig. S4). However, taxa belonging to the Bacteroidetes phylum tended to be high in NAFLD as well as in PC and did not discriminate between these two groups.

The Random Forest algorithm was used to construct a classification model based on the features discriminating PC from healthy controls, identified by LEfSe. As this algorithm’s performance greatly improves with larger sample size, all samples from the healthy, NAFLD, and PC groups were used for this analysis (n = 59), this time defining both NAFLD and healthy as “Control”. 70% (n = 41) of the samples were randomly chosen to train the classifier, and the remaining 18 samples were used for validation (Fig. 2b). In spite of the heterogenous nature of the Control group, an AUC value of 82.5% (CI: 63.6%-100%) was achieved for the validation set, with a specificity of 0.8 and sensitivity of 0.77.

### Additional clinical factors affecting the microbiome

As a number of the background conditions often found in PC patients have been previously shown to affect the microbiome, we proceeded to investigate whether PC-associated microbial patterns are dependent on additional clinical factors. First, we used the ANOSIM probability test to examine the effect of each of five clinical variables- bile duct obstruction, diabetes, hyperlipidemia, high blood pressure, and gall-bladder abnormalities- on the microbiome of PC patients. As shown in Supplementary Table S2, bile duct obstruction was the only variable to exert a significant, though minor, effect on the microbiome (p = 0.03, R = 13%, Bray-Curtis distance index; for the other clinical factors tested, p-values ranged from 0.2 to 0.8). To infer whether bile-duct obstruction was the main factor affecting PC microbiome, we repeated the Control vs. PC ANOSIM test of significance, using only samples from PC patients who had no bile duct obstruction (n = 16). Significant, though weak, separation between Control and non-obstructed PC was still observed (ANOSIM p = 0.01, R = 13%, Bray-Curtis index).

**Figure 3:**
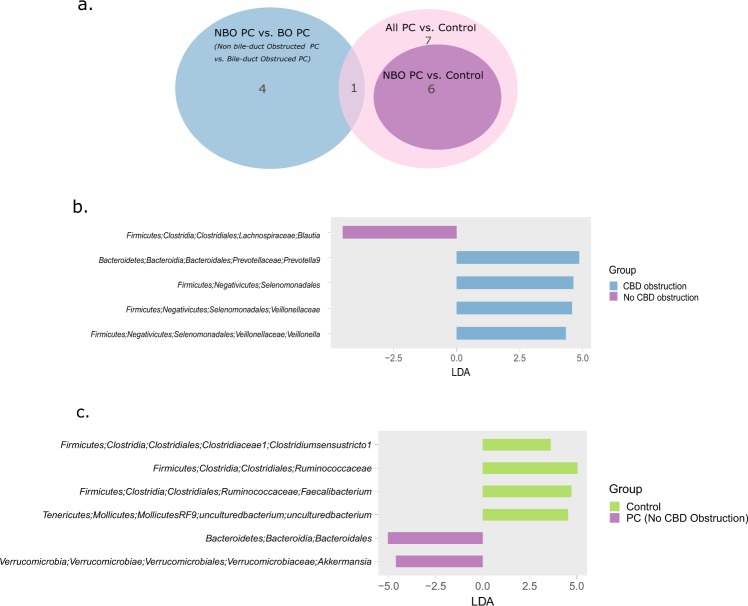
A different set of biomarker taxa is associated with PC and bile-duct obstruction. (**a**) Venn diagram showing the number of taxa shared between the three marker taxa sets identified in LEfSe comparisons. NBO PC: Non-Bile duct-Obstructed PC; BO PC: Bile duct-obstructed PC. (**b**) Taxa identified by LEfSe as differentiating between BO PC and NBO PC patients. (**c**) Taxa identified by LEfSe as differentiating between healthy controls and “pure”, NBO PC patients.

The LEfSe biomarker discovery tool was then used to identify which specific bacterial taxa contribute to the difference between bile-duct obstructed (BO, n = 11) vs. non bile-duct obstructed (NBO, n = 16) PC, as well as to the difference between healthy controls (n = 13) vs. NBO PC. Both these subsets of marker taxa could then be compared to the entire set of taxa that discriminates healthy controls from all PC (including both BO and NBO PC, shown in Fig. 2a). Of note, there was very little overlap between the PC-associated taxa and biliary-obstruction-associated taxa (Fig. 3a). Only 1 taxon, the Veillonellaceae family, was found to be associated with both biliary obstruction and PC; however, different genera of this family were associated with each condition: the genus *Veillonella* was associated with biliary obstruction (Fig. 3b), while the genus *Megasphaera* was associated with PC. Conversely, the set of taxa discriminating non-obstructed PC from controls recapitulated much of the all-PC vs Controls microbial signature (Fig. 3a,c).

### Additional microbiome changes may be a secondary consequence of PC

As bile duct obstruction usually leads to increased serum bilirubin levels (direct BL levels in unobstructed PC patients: 0.13 mg/dl, sd: 0.08; in obstructed patients: 2.6 mg/dl, median: 2.5; Kruskal-Wallis p = 0.004), we also examined correlations between bacterial genera and serum bilirubin levels (Supplementary Table S3). Interestingly, almost all (16/17) of the strongest correlations were negative, and mainly involved genera of the *Clostridiales* order (Spearman’s R = −0.5, p = 0.009 for the strongest correlation). The only *positive* correlation between bacteria and bilirubin was found for *Prevotella9*, the same genus we had identified as biliary-obstruction-associated in the section “**Additional clinical factors affecting the microbiome**” (Spearman’s R = −0.36, p = 0.065). However, these results did not reach statistical significance following p-value correction for multiple hypothesis testing (best q-value 0.23, FDR^24^). A similar profile, yet more statistically robust, was obtained when correlating the genera to the GGT enzyme serum levels, an additional, often earlier, marker of biliary obstruction (Table 2).

**Table 2:** Bacterial genera correlating with serum GGT levels in PC patients.

Taxon	R	p	FDR
Firmicutes;Clostridia;Clostridiales;Ruminococcaceae;Subdoligranulum	−0.626	0.001	0.038
Proteobacteria;Alphaproteobacteria;Rhodospirillales;Rhodospirillaceae;uncultured	−0.651	0.001	0.038
Firmicutes;Clostridia;Clostridiales;Christensenellaceae;Christensenellaceae R-7 group	−0.563	0.004	0.103
Proteobacteria;Deltaproteobacteria;Desulfovibrionales;Desulfovibrionaceae;Bilophila	−0.515	0.01	0.192
Actinobacteria;Actinobacteria;Bifidobacteriales;Bifidobacteriaceae;Bifidobacterium	−0.42	0.041	0.21
Bacteroidetes;Bacteroidia;Bacteroidales;Porphyromonadaceae;Butyricimonas	−0.424	0.039	0.21
**Bacteroidetes;Bacteroidia;Bacteroidales;Prevotellaceae;Prevotella 9**	**0**.**466**	**0**.**022**	**0**.**21**
Firmicutes;Clostridia;Clostridiales;Lachnospiraceae;Anaerostipes	−0.429	0.036	0.21
Firmicutes;Clostridia;Clostridiales;Lachnospiraceae;Blautia	−0.453	0.026	0.21
Firmicutes;Clostridia;Clostridiales;Lachnospiraceae;Fusicatenibacter	−0.423	0.04	0.21
Firmicutes;Clostridia;Clostridiales;Lachnospiraceae;Lachnospiraceae NK4A136 group	−0.485	0.016	0.21
Firmicutes;Clostridia;Clostridiales;Lachnospiraceae;[Ruminococcus] torques group	−0.43	0.036	0.21
Firmicutes;Clostridia;Clostridiales;Ruminococcaceae;Ruminiclostridium 5	−0.465	0.022	0.21
Firmicutes;Clostridia;Clostridiales;Ruminococcaceae;Ruminococcaceae UCG-014	−0.464	0.022	0.21
Firmicutes;Erysipelotrichia;Erysipelotrichales;Erysipelotrichaceae;Holdemanella	0.42	0.041	0.21

Spearman’s correlation method was used, followed by FDR-based p-value adjustment, Correlations with q < = 0.21 are shown.

As pancreatic cancer and bile-duct obstruction are often associated also with elevated liver enzyme levels, we went on to correlate microbial composition with serum levels of liver enzymes, serving as markers of impaired liver function. Both AST and ALT were available for the PC patients in this cohort. As we found the 2 markers to be strongly inter-correlated (Pearson’s R = 0.9, p = 5.4E-11), and since we observed similar microbial/enzyme-levels associations across both assays, only the correlations to ALT are shown (Table 3).

**Table 3:** Bacterial genera correlating with serum ALT levels in PC patients.

Taxon	R	p	FDR
Proteobacteria;Alphaproteobacteria;Rhodospirillales;Rhodospirillaceae;uncultured	−0.692	<0.001	<0.001
Firmicutes;Clostridia;Clostridiales;Christensenellaceae;Christensenellaceae R-7 group	−0.582	0.003	0.077
Firmicutes;Clostridia;Clostridiales;Ruminococcaceae;Subdoligranulum	−0.579	0.003	0.077
**Bacteroidetes;Bacteroidia;Bacteroidales;Prevotellaceae;Prevotella 9**	**0**.**527**	**0**.**008**	**0**.**139**
Firmicutes;Clostridia;Clostridiales;Ruminococcaceae;Ruminococcaceae UCG-014	−0.524	0.009	0.139
Firmicutes;Clostridia;Clostridiales;Ruminococcaceae;Ruminiclostridium 5	−0.474	0.019	0.209
Proteobacteria;Deltaproteobacteria;Desulfovibrionales;Desulfovibrionaceae;Bilophila	−0.479	0.018	0.209

Spearman’s correlation method was used, followed by FDR-based p-value adjustment, Correlations with q < = 0.21 are shown.

The correlation profile of liver-damage-markers to microbiome mirrors that observed for biliary-obstruction markers to microbiome. All the strongest associations between relative abundance of microbial taxa and liver-enzyme serum-levels were negative (Table 3), with the sole exception of *Prevotella 9*. As bacterial composition is based on relative abundances, a large increase in one taxon may be mistakenly interpreted as reductions in other taxa. To account for this possibility, we repeated the correlation analysis after removing *Prevotella 9* from the data and recalculating relative abundances for all other taxa, in its absence. This procedure had almost no effect on the correlation analysis results (Rho values for the significantly correlated taxa changed by less than 5%). Taken together, these data suggest PC-associated liver-damage disrupts the normal gut equilibrium, driving reductions in multiple normal gut-residing bacteria.

Liver-assay tests and bilirubin levels were also available for the cohort of non-alcoholic fatty liver patients (n = 16), enabling analysis of microbiome/functional-markers interactions independent of a PC status. It should be noted that for fatty-liver patients, the inter-patient variation of all five biochemical markers was much lower than among the PC patients (Supplementary Table S4). That being said, microbiome associations with biliary obstruction markers (bilirubin and GGT) were still observed, but of a different nature then in PC patients. Specifically, only one taxon was correlated to GGT (*Lachnoclostridium*, R = −0.764, p = 0.001, q = 0.077). Several taxa were correlated to bilirubin (Table 4); but, while in PC patients we observed multiple negative reductions across the Clostridiales order, in fatty-liver patients only the genus *Dialister*, of the *Selemondales* order, was negatively correlated to bilirubin. Conversely, we observed three positive microbial/bilirubin correlations in the fatty-liver group, none of them to *Prevotella 9*. No correlations to either AST or ALT were found in the fatty-liver patient group.

**Table 4:** Bacterial genera correlating with bilirubin levels in fatty liver patients.

Taxon	Rho	p	FDR
Firmicutes;Negativicutes;Selenomonadales;Veillonellaceae;Dialister	−0.822	<0.001	<0.001
Bacteroidetes;Bacteroidia;Bacteroidales;Rikenellaceae;Alistipes	0.737	0.001	0.026
Firmicutes;Negativicutes;Selenomonadales;Acidaminococcaceae;Phascolarctobacterium	0.764	0.001	0.026
Firmicutes;Clostridia;Clostridiales;Ruminococcaceae;Ruminiclostridium 6	0.632	0.009	0.173

Spearman’s correlation method was used, followed by FDR-based p-value adjustment, Correlations with q < = 0.21 are shown.

### Intra-cohort variation

To explore possible diagnostic value of PC-associated microbial patterns, we focused on 10 bacterial genera that had been identified in LEfSe analysis as being either under-represented or overrepresented in PC when compared to healthy controls (shown in section “**Specific microbial patterns associated with PC”**, Fig. 2). We closely examined these potential biomarker genera in a broader context, comparing their abundance patterns across multiple subject groups - the NAFLD subject group (n = 16), and a pre-cancerous pancreatic lesions group (PCL, n = 6), as well as our original PC and healthy control groups. From this perspective, promising biomarkers should show a similar trend for PC and PCL, when compared to both healthy and fatty-liver control groups. To address consistency issues, we also examined the relative abundance of each of these discriminatory genera across each of the subjects in our cohort **(**for summary boxplots across the PC, PCL, NAFLD and healthy groups, as well as per-subject barplots, for each of the PC discriminating genera, see Supplementary Fig. S5).

**Figure 4:**
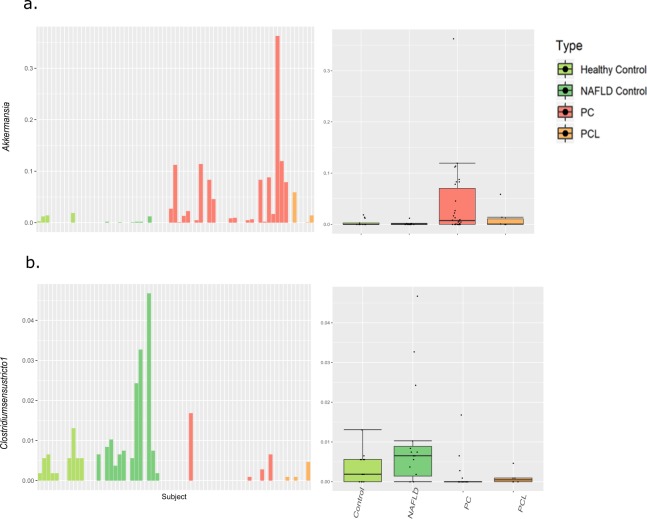
In-depth exploration of two representative PC discriminating taxa. A boxplot, portraying summary statistics across the different groups, and a corresponding barplot, portraying the relative abundance in each individual, are shown for the PC-associated *Akkermansia* (**a**), and the control-associated *Clostridium sensu stricto 1*. (**b**) In the boxplots, lines indicate median relative abundances, boxes denote 3^rd^ and 1^st^ quantile, whiskers denote ± 1.5*IQR, and outliers are shown as dots.

For the most part, the genera we examined showed the same trend in the NAFLD control group as in the healthy control group. The exceptions were *Megasphaera* and *Lachnospiraceae UCG_008*, both of which were overrepresented in NAFLD as well as in PC. However, most potential biomarker genera did not display similar trends in the PCL and PC groups. Furthermore, all of the discriminatory taxa were highly variable between individuals, across all subject groups. These trends are illustrated in Fig. 4, using as an example two of the discriminatory genera: *Akkermansia*, in our dataset associated with PC (Fig. 4a); and *Clostridium sensu stricto 1*, in our dataset associated with healthy subjects (Fig. 4b).

### Inter-cohort comparisons

These results mirror, to a certain extent, those recently published by Ren *et al*.^10^ in a similar study comparing PC patients and controls in a Chinese cohort. Both cohorts showed a similar trend at the phylum level, with an increase of Bacteroidetes and a decrease of Firmicutes in PC patients. This similarity extended, in part, to finer taxonomic levels; Anaerostipes and genera belonging to *Erysipelotrichaeceae* and *Clostridiaceae* decreased in PC, while genera belonging to *Veillonellaceae* increased in PC, across both cohorts.

**Figure 5:**
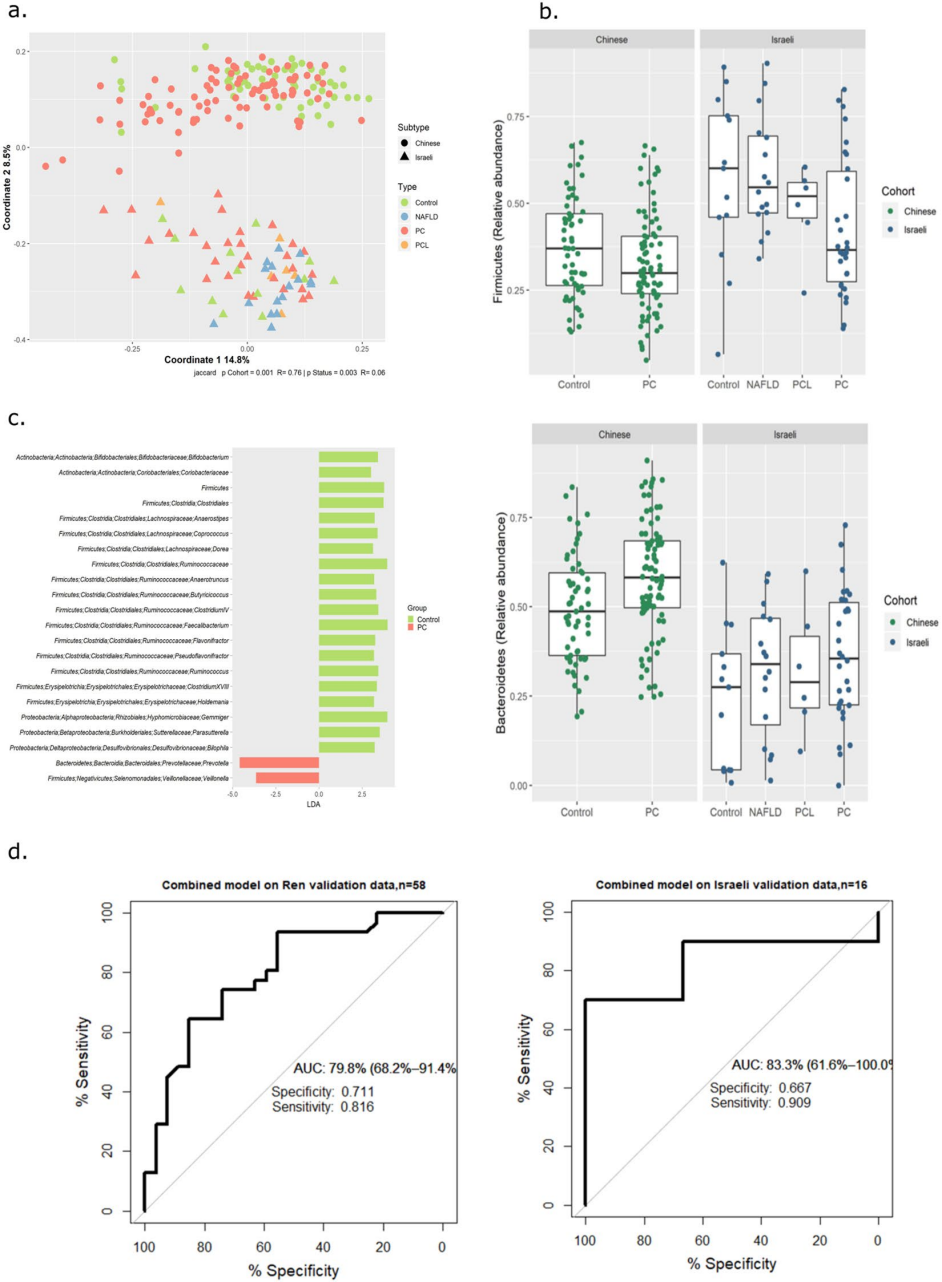
Both origin and disease effects are evident in an integrative analysis of two cohorts. Data from a Chinese cohort^10^ was integrated with our own to form a merged dataset for analysis. (**a**) PCoA of Jaccard distance matrix. (**b**) Boxplots of key phyla across cohort and type. (**c**) LEfSe analysis identifies disease discriminating taxa in the integrated dataset. (**d**) ROC of a random forest model built on the features identified in (c). In the boxplots, lines indicate median relative abundances, boxes denote 3^rd^ and 1^st^ quantile, whiskers denote ± 1.5*IQR, and outliers are shown as dots.

To explore inter-cohort similarities and differences in greater detail, we merged our data with data publicly available from the Ren study (Methods section), forming a uniform dataset for analysis. Notably, sample origin (Israeli or Chinese) exerted the strongest effect on sample composition (Fig. 5a). This effect is driven partly by differential representation of the less-common gut taxa across the two cohorts (39 taxa were unique to the Chinese cohort, and 63 to the Israeli cohort); and partly by highly increased abundance of taxa belonging to the Bacteroidetes phylum, and decreased abundance of the Firmicutes phylum, across the entire Chinese cohort compared to the Israeli one (p < e^−6^). Due to this latter feature, when the Random Forest classifier that was trained on our data was applied to the Chinese data, all of the Chinese samples (both PC *and* Control) were classified as PC. Nevertheless, *within* each cohort, similar PC-associated patterns were clearly observed (Fig. 5b). A Random Forest classifier built on the integrated dataset (by first identifying the most relevant features for this dataset using LEfSe, Fig. 5c) was able to classify both Israeli and Chinese samples with a specificity of 0.66 and 0.71, respectively, and a sensitivity of 0.9 and 0.81 respectively (Fig. 5d). These values are not as high as those obtained for each dataset alone, but are much better than random, and support the view that PC-specific dysbiosis is evident in two independent human populations.

## Discussion

The advancements in molecular methods for microbiome characterization over the past decade have spurred an expansion of studies exploring the composition of the human gut microbiome. Taken collectively, these studies show the gut microbiome to be a complex environment, affected by numerous genetic and environmental factors^11,27,28^. While the microbiome greatly varies between individuals, most of this variation falls within the scope of what might be termed a “healthy gut microbiome”. Microbial dysbiosis, or a deviation from this equilibrium, has been described for a variety of disease, among them diabetes^29^, liver cirrhosis^30^, and inflammatory bowel disease^31^.

In this study we find a distinct PC-associated gut microbiome signature in an Israeli cohort, manifesting primarily as an under-representation in several bacterial families prevalent in the healthy gut - *Clostridiacea*, *Lachnospiraceae*, and *Ruminococcaceae;* and an over-representation of *Veillonellaceae*, *Akkermansia* and *Odoribacter*. This signature was distinct from that of common PC co-morbidities which also affected the microbiome, such as bile-duct obstruction and liver damage.

Several of the PC-associated microbial characteristics of our cohort were similar to those of a previous study conducted on a Chinese cohort^10^. When integrating both datasets to a combined analysis, we found extensive differences in microbial composition between the Israeli and Chinese cohorts. These differences are probably driven not only by diet and ethnic origin, but also by differences in methodology; in particular, the different DNA extraction methods that were used. Nevertheless, the existence of similar trends across both these cohorts suggests many PC-associated microbial patterns are highly robust.

One of the promising aspects of microbiome research is the possibility of utilizing microbial patterns for development of diagnostic tools, based on bacterial “biomarkers”. However, translation of a disease-associated microbial signature into effective diagnostic biomarkers is not straightforward, and must address potentially confounding issues, such as the inherent variability between cohorts of different origin as well as between individuals within each cohort. The strong effect of origin we found in our integrated cohort analysis illustrates that specific classifiers should be trained for specific patient populations. However, even in the “best” case, the specificity of our microbial classifiers was between 0.7 to 0.8; similar values were reported by Ren *et al*. For a low-incidence disease such as PC (with an odds ratio of ~1:20000), these specificity values would translate to approximately 4,000 false positives per each true diagnosis. The disparity between existence of robust disease-associated features identified by LDA, and the limited classification power of these same features, probably stems from the high variability of microbial features across individuals.

As late-stage PC is almost impossible to treat, a vital criterion for PC biomarkers is recognition of the disease at a very early stage. A major limitation of this study was the small size of the PCL group, which did not provide sufficient statistical power. Future studies enrolling larger numbers of subjects with pre-cancerous lesions or very early-stage PC may thus reveal additional microbial patterns. However, the effects of PC on gut microbiome are probably mediated primarily via alterations in pancreatic exocrine secretions to the digestive system, and therefore are expected to increase as tumor size increases. Thus, the difficulties we encountered in translating robust microbial patterns to actual PC classification are likely to be even more extensive when attempting to identify early-stage PC. However, a feasible approach may be to combine several microbial features with other non-invasive biomarkers, such as the serum biomarker CA19-9 which is of limited use in PC detection, or urinary biomarkers currently being investigated^32^, for increased accuracy.

### Summary

A distinct PC-associated fecal microbiome signature, resembling findings previously reported in a Chinese cohort, was observed in this study. However, given the low incidence of PC and the high variability in microbiome both within and between the cohorts, harnessing microbial patterns for diagnostic purposes may only be practical if combined with additional biomarkers.

## Supplementary information

Supplementary Figures

Supplementary Tables

## Data Availability

The datasets generated during and analyzed during the current study are available from the corresponding author on reasonable request. All sequence data is available from NCBIs Sequence Read Archive (SRA), BioProject ID: PRJNA575620.
