# Supplementary material for: Fecal microbiome signatures of pancreatic cancer patients
Source: Sci Rep. 2019 Nov 14;9:16801. doi: 10.1038/s41598-019-53041-4 (PMC6856127; doi:10.1038/s41598-019-53041-4)
Supplement: Supplementary file 1 — Supplementary Figures [file 41598_2019_53041_MOESM1_ESM.pdf]

Fecal microbiome signatures of pancreatic cancer patients

Elizabeth Half[1]*, Nirit Keren[2]*, Leah Reshef[2], Tatiana Dorfman[3], Ishai Lachter[1], Yoram Kluger[3], Naama Reshef[4], Hilla Knobler[4], Yaakov Maor[5], Assaf Stein[6], Fred M Konikoff[6,7], and Uri Gophna**[2]

[1]Department of Gastroenterology, Rambam Health Care Campus; [2]Department of Molecular Microbiology and Biotechnology, George S. Wise Faculty of Life Sciences; [3]Department of General Surgery, Rambam Health Care Campus; [4]Diabetes, Metabolic and Endocrinology Institute, Kaplan Medical Center, Rehovot, Israel; [5]Institute of Gastroenterology and Hepatology. Kaplan Medical Center, Rehovot; [6]Department of Gastroenterology and Hepatology, Meir Medical Center, Kfar Saba; [7]Sackler Faculty of Medicine, Tel Aviv University, Israel

*Equal Contributors

A.

B.

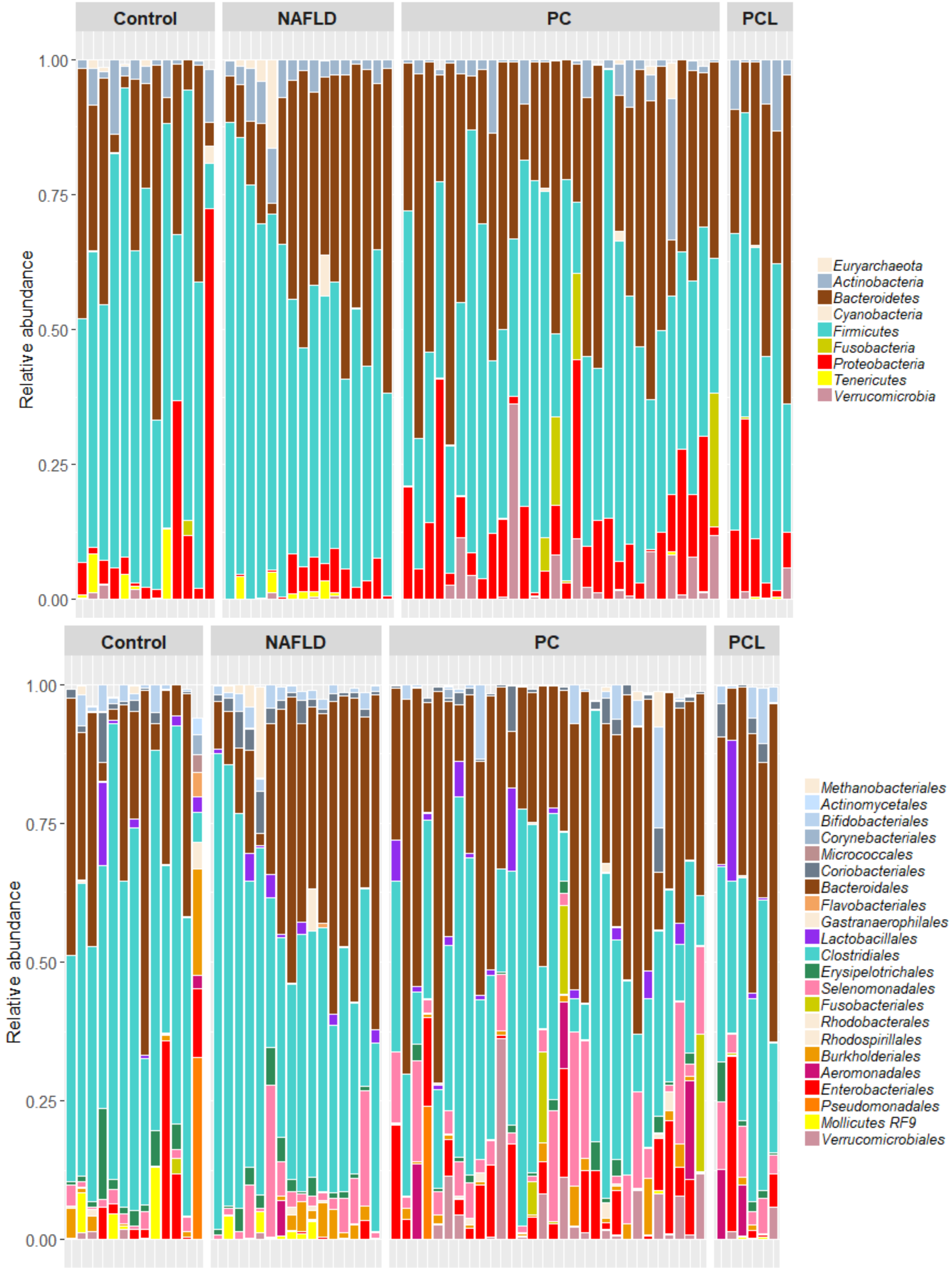

Figure S1. Full taxonomic profiles of all samples used in this study.
A. Phyla level; B. Order level; C. Family level; D. Genus level. Taxa whose relative abundance was below 3% across all the samples are not shown. The main phyla, orders and families of the gut are color coded: genera, families and orders of the Bacteroidetes are in shades of brown; genera and families of the *Clostridiales* order (belonging to the Firmicutes phylum) are in shades of blue and green.

C.

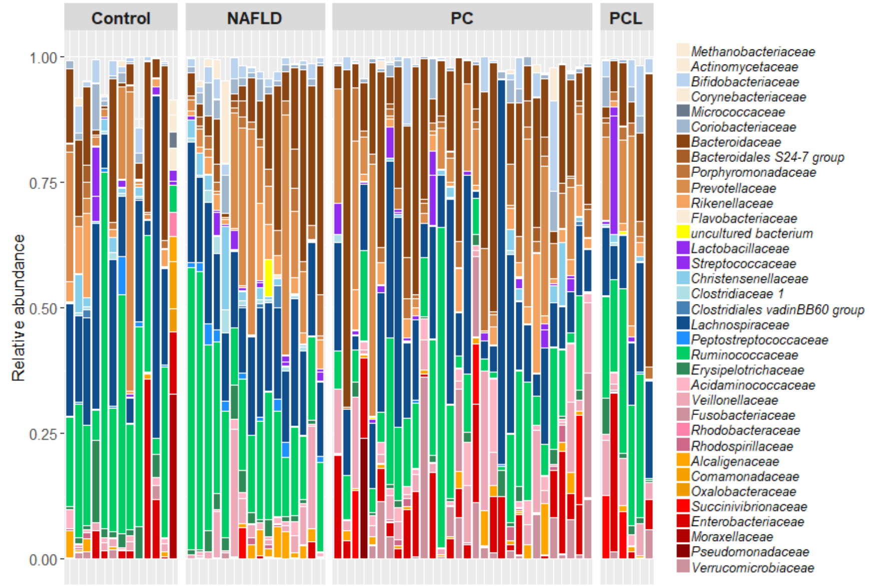
Control
NAFLD
PC
PCL
1.00
0.75
0.50
0.25
0.00
Relative abundance
Methanobacteriaceae
Actinomycetaceae
Bifidobacteriaceae
Corynebacteriaceae
Micrococcaceae
Coriobacteriaceae
Bacteroidaceae
Bacteroidales S24-7 group
Porphyromonadaceae
Prevotellaceae
Rikenellaceae
Flavobacteriaceae
uncultured bacterium
Lactobacillaceae
Streptococcaceae
Christensenellaceae
Clostridiaceae 1
Clostridiales vadinBB60 group
Lachnospiraceae
Peptostreptococcaceae
Ruminococcaceae
Erysipelotrichaceae
Acidaminococcaceae
Veillonellaceae
Fusobacteriaceae
Rhodobacteraceae
Rhodospirillaceae
Alcaligenaceae
Comamonadaceae
Oxalobacteraceae
Succinivibrionaceae
Enterobacteriaceae
Moraxellaceae
Pseudomonadaceae
Verrucomicrobiaceae

D.

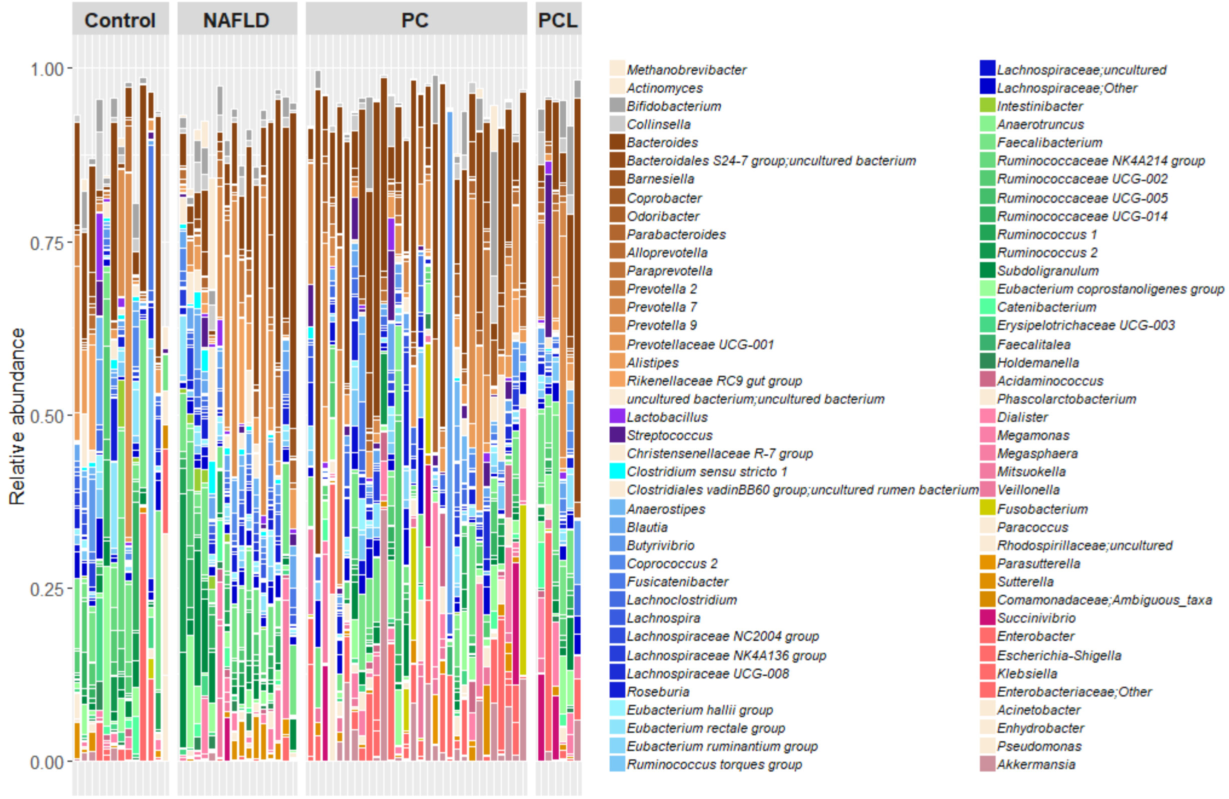
Control
NAFLD
PC
PCL
1.00
0.75
0.50
0.25
0.00
Relative abundance
Methanobrevibacter
Actinomyces
Bifidobacterium
Collinsella
Bacteroides
Bacteroidales S24-7 group;uncultured bacterium
Barnesiella
Coprobacter
Odoribacter
Parabacteroides
Alloprevotella
Paraprevotella
Prevotella 2
Prevotella 7
Prevotella 9
Prevotellaceae UCG-001
Alistipes
Rikenellaceae RC9 gut group
uncultured bacterium;uncultured bacterium
Lactobacillus
Streptococcus
Christensenellaceae R-7 group
Clostridium sensu stricto 1
Clostridiales vadinBB60 group;uncultured rumen bacterium
Anaerostipes
Blautia
Butyrivibrio
Coprococcus 2
Fusicatenibacter
Lachnoclostridium
Lachnospira
Lachnospiraceae NC2004 group
Lachnospiraceae NK4A136 group
Lachnospiraceae UCG-008
Roseburia
Eubacterium hallii group
Eubacterium rectale group
Eubacterium ruminantium group
Ruminococcus torques group
Lachnospiraceae;uncultured
Lachnospiraceae;Other
Intestinibacter
Anaerotruncus
Faecalibacterium
Ruminococcaceae NK4A214 group
Ruminococcaceae UCG-002
Ruminococcaceae UCG-005
Ruminococcaceae UCG-014
Ruminococcus 1
Ruminococcus 2
Subdoligranulum
Eubacterium coprostanoligenes group
Catenibacterium
Erysipelotrichaceae UCG-003
Faecalitalea
Holdemanella
Acidaminococcus
Phascolarctobacterium
Dialister
Megamonas
Megasphaera
Mitsuokella
Veillonella
Fusobacterium
Paracoccus
Rhodospirillaceae;uncultured
Parasutterella
Sutterella
Comamonadaceae;Ambiguous_taxa
Succinivibrio
Enterobacter
Escherichia-Shigella
Klebsiella
Enterobacteriaceae;Other
Acinetobacter
Enhydrobacter
Pseudomonas
Akkermansia

Figure S2. Basic microbiome characteristics across all patient groups. A. Microbial diversity (alpha diversity, measured by Shannon index); B. The ratio between total abundance of the Bacteroidetes phylum to total abundance of the Firmicutes phylum. Dunns test was used for to test for significance across all groups; significant (p<0.05) and borederline-significant (p<0.1) results are marked in bold.

A.

Comparisons	Z statistic	adjusted P value
**Control - FattyLiver**	**-1.663622469**	**0.048093949**
Control - IPMN	0.002747691	0.498903831
FattyLiver - IPMN	1.300449415	0.096723492
Control - PC	0.545634602	0.292658567
**FattyLiver - PC**	**2.591869416**	**0.0047728**
IPMN - PC	0.402092429	0.343808002

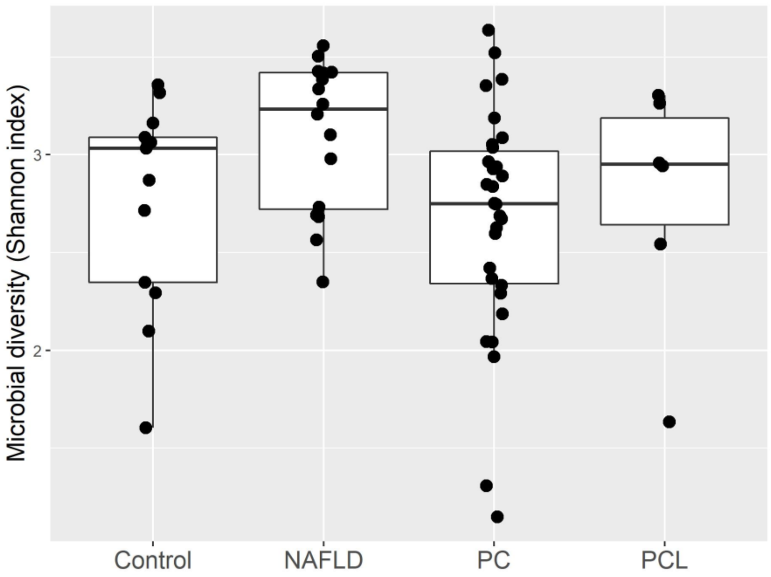

B.

Comparisons	Z statistic	adjusted P value
Control - NAFLD	-0.705490331	0.240252574
**Control - PC**	**-2.213577502**	**0.013428928**
**NAFLD - PC**	**-1.523372851**	**0.063832725**
Control - PCL	-0.685548872	0.246498816
NAFLD - PCL	-0.156514266	0.437813838
PC - PCL	0.886968594	0.187547905

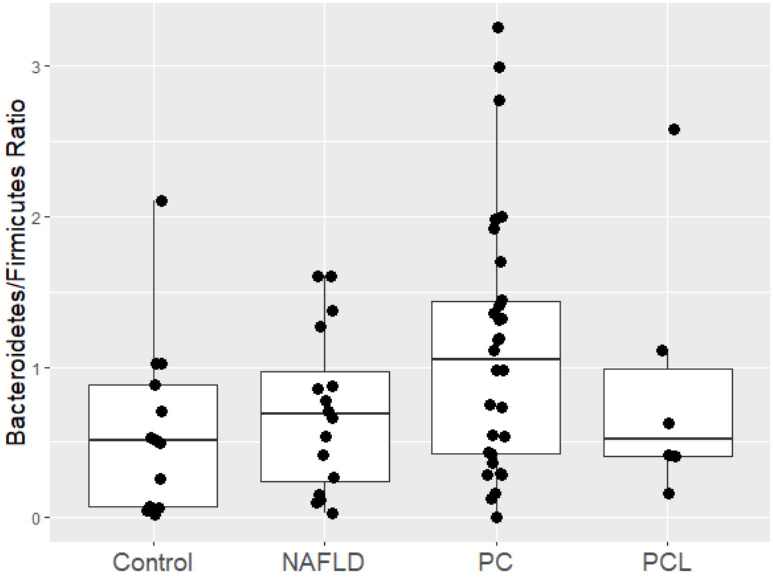

Figure S3. Heirarchial clustering of all samples according to similarity in microbial composition. Distance matrices based on either abundance-weighted (left) or unweighted (right) UniFrac matrices . UPGMA method was used for clustering; dendrogram labels are color-coded according to the participants clinical status, as in Figures 1 and 4.

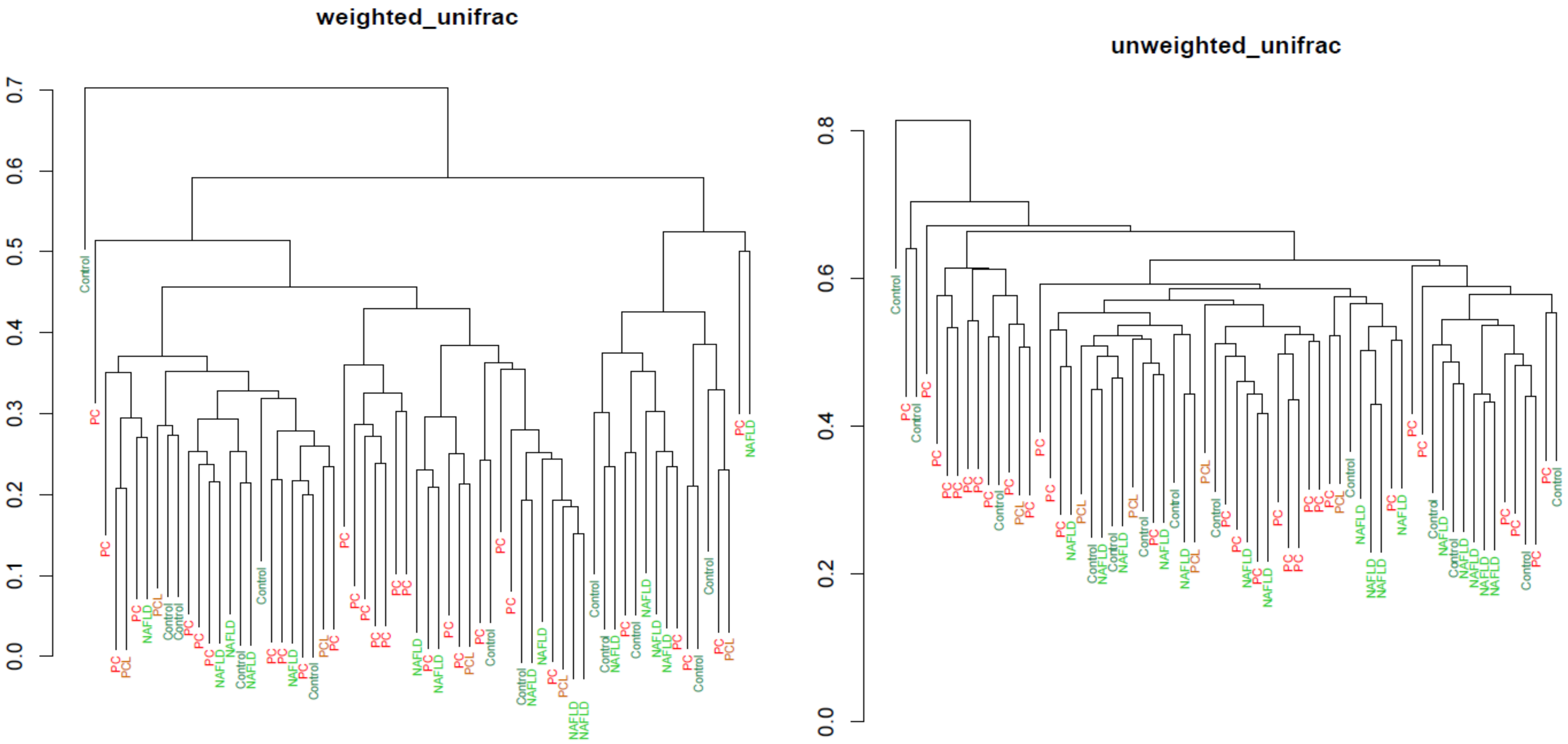

Figure S4.Bacterial taxa identified by LEfSe as differentiating between PC patients (red) and NAFLD control subjects (green).

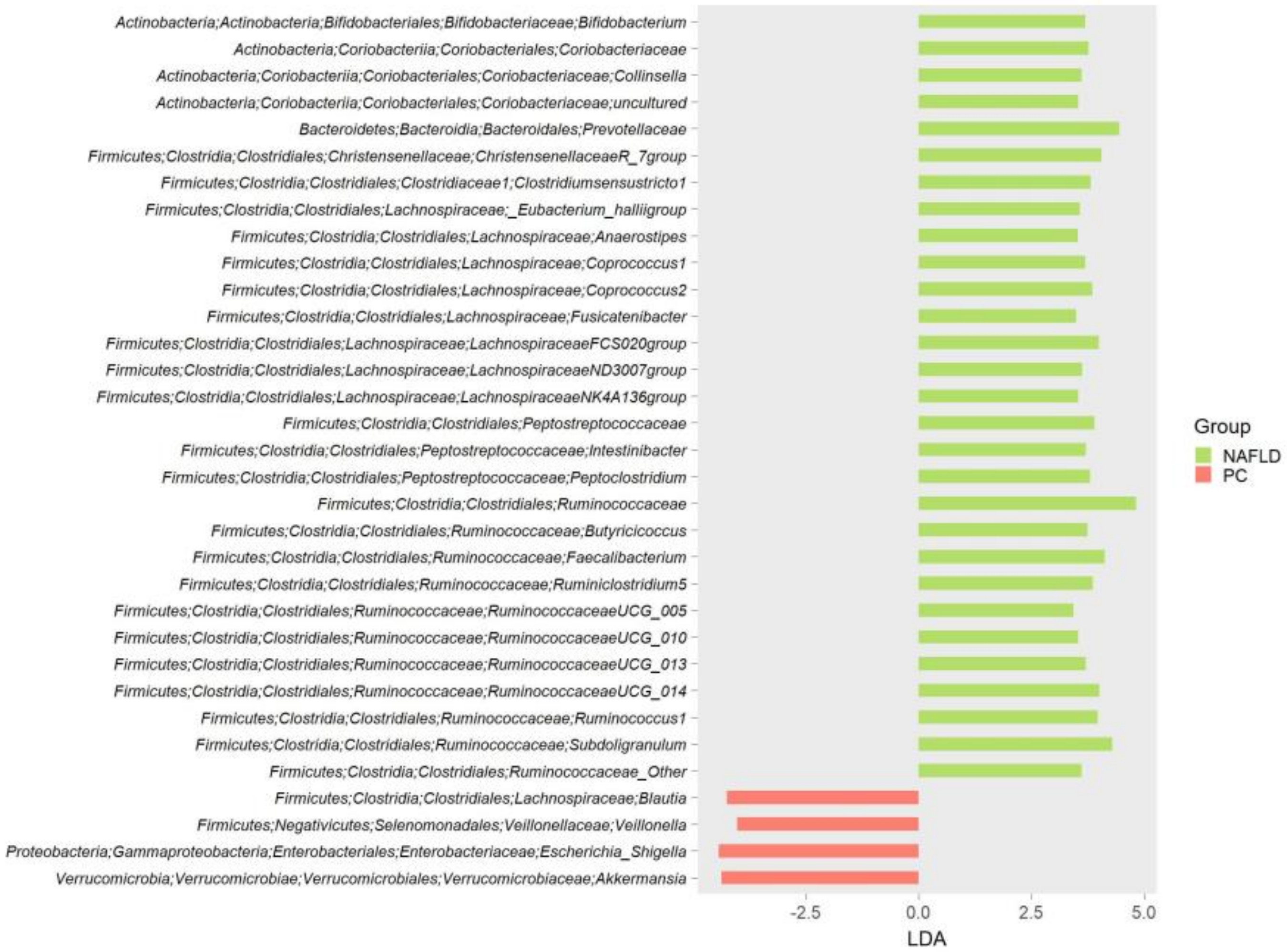

Figure S5. Inter-group and intra-group patterns of all genera dentified by LEfSe as differentiating between PC patients and health controls.

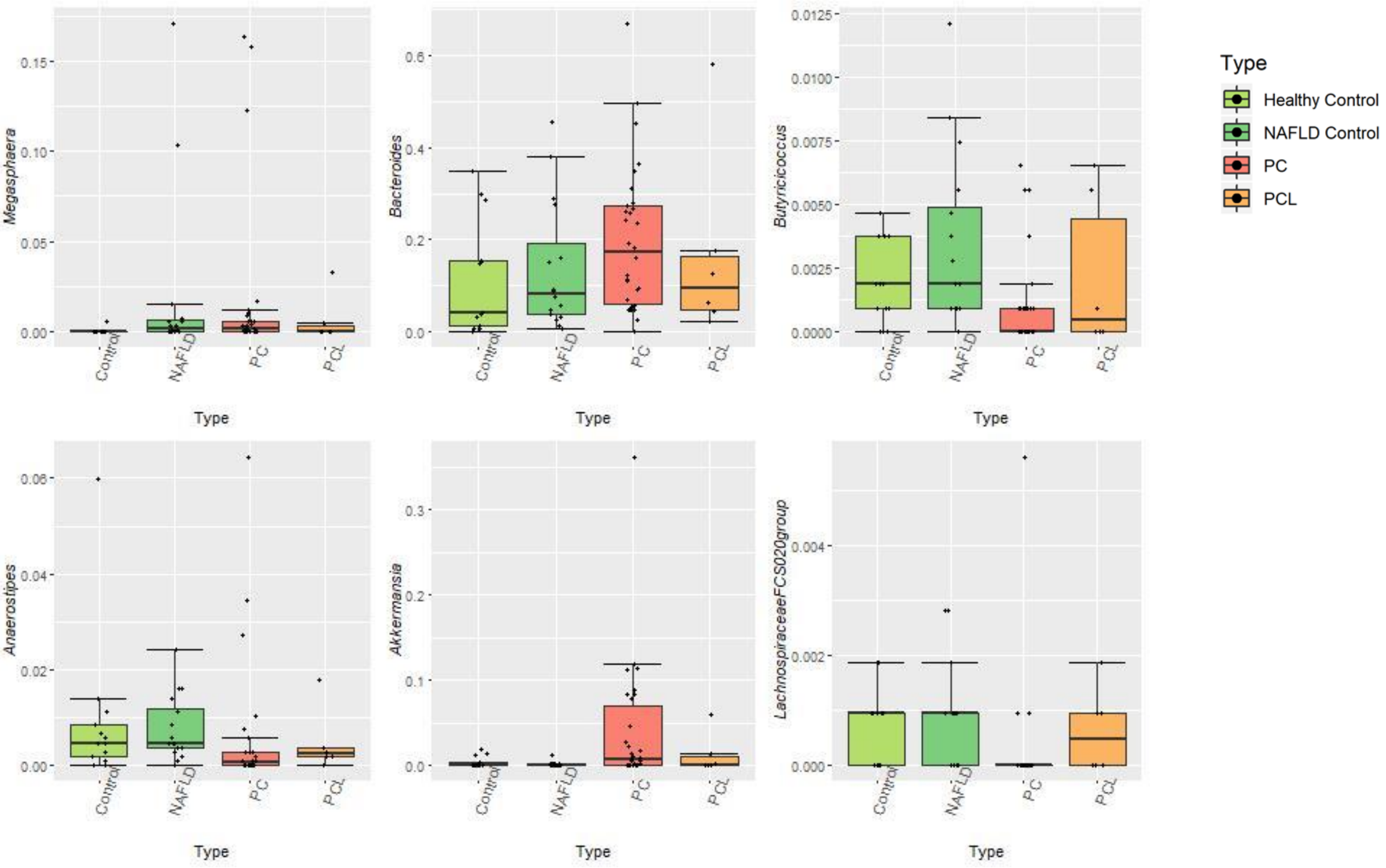

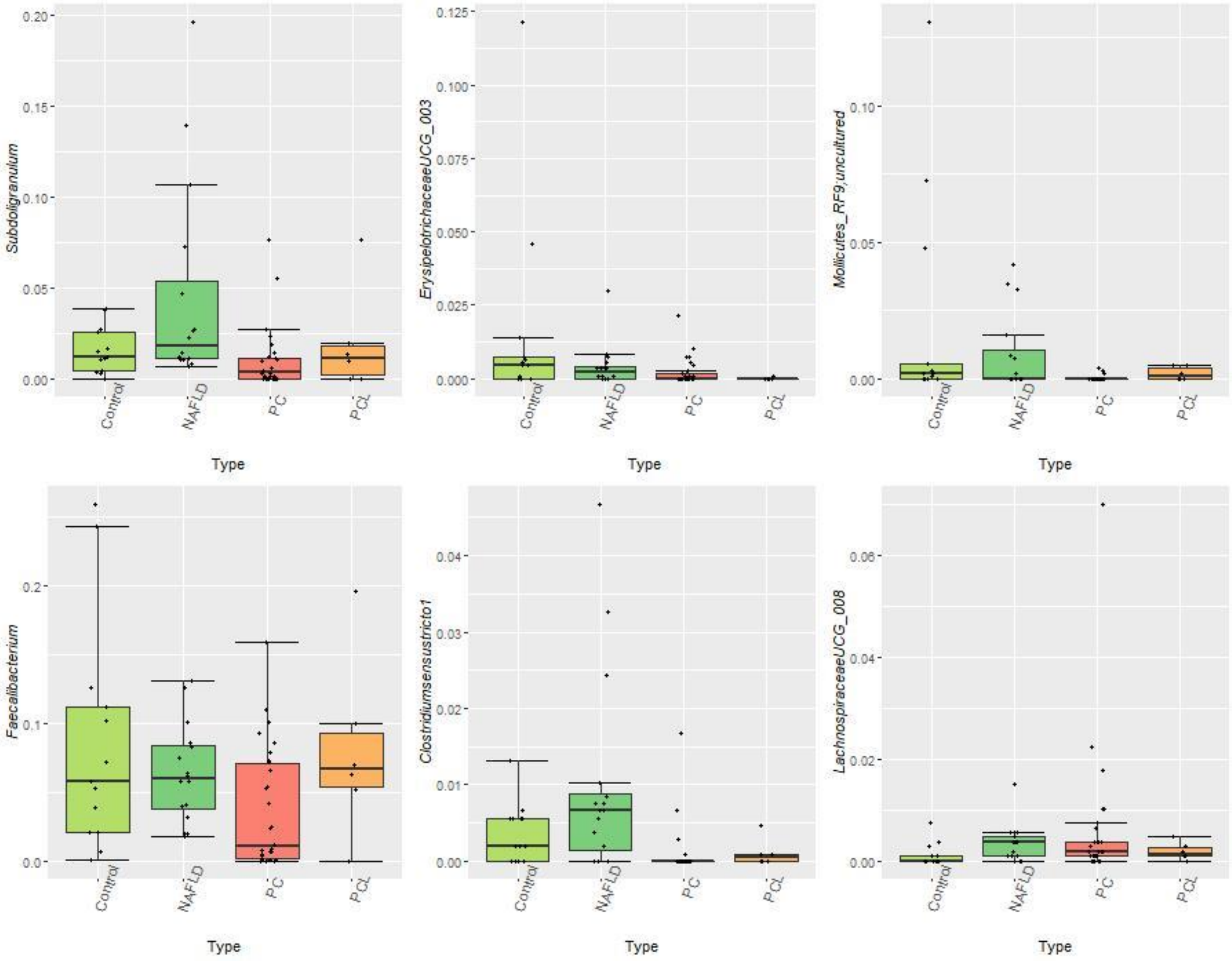

Subdoligranulum
ErysipelotrichaceaeUCG_003
Mollicutes_RF9;uncultured
Faecalibacterium
Clostridiumsensustricto1
LachnospiraceaeUCG_008
Control
NAFLD
PC
PCL
Type

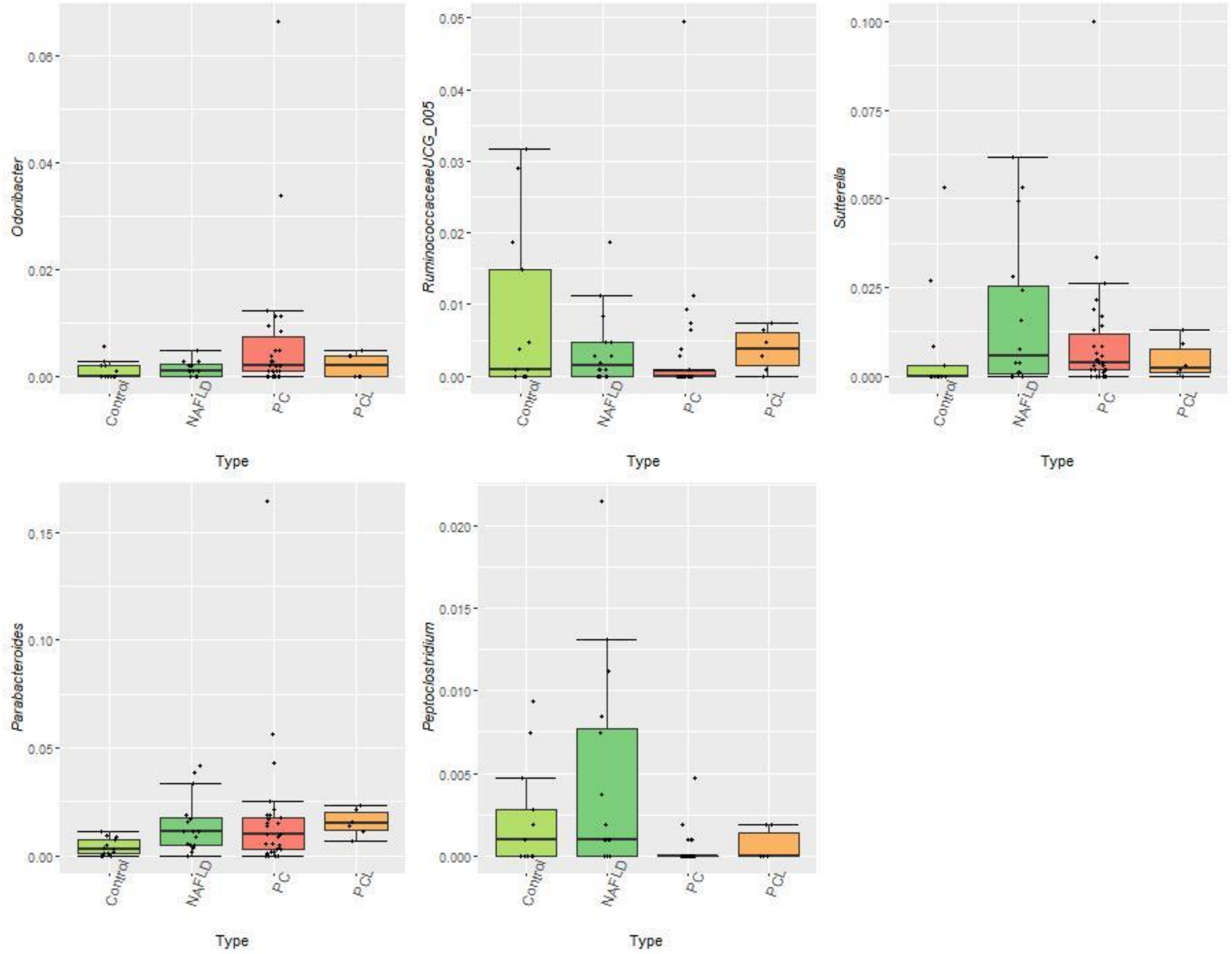
Odoribacter
0.00
0.02
0.04
0.06
Control
NAFLD
PC
PCL
Type
RuminococcaceaeUCG_005
0.00
0.01
0.02
0.03
0.04
0.05
Control
NAFLD
PC
PCL
Type
Sutterella
0.000
0.025
0.050
0.075
0.100
Control
NAFLD
PC
PCL
Type
Parabacteroides
0.00
0.05
0.10
0.15
Control
NAFLD
PC
PCL
Type
Peptoclostridium
0.000
0.005
0.010
0.015
0.020
Control
NAFLD
PC
PCL
Type
